# Dynamics of Carbon Storage Allocation and Its Drivers in Post-Fire *Quercus acutissima* Forests During Successional Recovery

**DOI:** 10.3390/plants15162525

**Published:** 2026-08-20

**Authors:** Yuhua Ma, Kang Liu, Hao Yu, Shuai Ma, Haotian Zhu, Yichen Fan, Cheng Huang, Fasih Ullah Haider, Xu Li, Chun Feng, Zhen Wu

**Affiliations:** 1 School of Modern Agriculure and Food Engineering, Chuzhou University, Chuzhou 239000, China; myh@chzu.edu.cn (Y.M.);; 2 Anhui Provincial Key Laboratory of Forest Resources and Silviculture, Anhui Agricultural University, Hefei 230036, China; 3 School of Intelligent Construction and Hydraulic Engineering, Chuzhou University, Chuzhou 239000, China; 4 Hubei Key Laboratory of Biologic Resources Protection and Utilization, Hubei Minzu University, Enshi 445000, China; 2024102@hbmzu.edu.cn; 5 Guangdong Provincial Key Laboratory of Applied Botany, South China Botanical Garden, Chinese Academy of Sciences, Guangzhou 510650, China; haider281@scbg.ac.cn (F.U.H.);; 6 School of Forestry and Landscape Architecture, Anhui Agricultural University, Hefei 230036, China; 7 School of Geographic Engineering and Spatial Information, Chuzhou University, Chuzhou 239000, China

**Keywords:** carbon sequestration capacity, ecological restoration, forest ecosystem, stand age

## Abstract

Post-fire plantations play a crucial role in recovering carbon stocks, yet how carbon is partitioned among vegetation, litter, and soil pools during stand growth dynamics remains insufficiently resolved for *Quercus acutissima* plantations. Forest ecosystems play a crucial role in the global carbon cycle. This study quantified carbon-storage allocation and identified stand characteristics and soil factors associated with carbon recovery in fire-affected *Q. acutissima* plantations. Using a chronosequence design, we compared five stand-age classes (4, 10, 25, 45, and 50 years) on Huangfu Mountain, China, and measured carbon stocks in tree organs, understory vegetation, litter, and the 0–30 cm soil layer. Ecosystem carbon stock increased from 31.64 t ha^−1^ in 4-year-old stands to 230.66 t ha^−1^ in 50-year-old stands, representing a 629% increase. Soil was the dominant carbon pool, with 0–30 cm soil carbon rising from 25.32 to 126.56 t ha^−1^ (a 400% increase). The contribution of soil carbon to total ecosystem storage declined from approximately 80% in 4-year-old stands to 55% in 50-year-old stands, indicating a shift in allocation toward vegetation biomass over time. Carbon accumulation was primarily concentrated in the 0–10 cm layer. Tree basal area was significantly associated with ecosystem carbon stocks, identified as a key structural factor linked to carbon accumulation through potential direct and indirect pathways involving light availability and soil carbon. Soil organic matter and nitrogen were also positively correlated with carbon accumulation. These findings suggest that stand development and topsoil carbon formation are closely linked to post-fire carbon recovery processes. Future management measures should optimize stand density, maintain soil fertility, and protect surface carbon to enhance long-term carbon sequestration.

## 1. Introduction

Forest ecosystems are among the largest terrestrial ecosystems, characterized by complex structures and multiple functions [1,2,3]. They play a crucial role in maintaining biodiversity and regulating the global carbon balance [4,5]. Forest degradation, resulting from rapid economic development, intensive exploitation of forest resources, and frequent natural disturbances, is a major global environmental issue [6,7]. Disturbances such as wildfires and logging alter forest age structure, species composition, and regeneration, ultimately initiating secondary succession [2,8]. In degraded forest ecosystems following disturbance, vegetation recovery serves as the foundation for restoring forest ecological functions and rebuilding ecosystem carbon stocks. Disturbances can reduce forest canopy closure, creating gaps that alter light availability and the growth environment, consequently affecting the entire forest ecosystem [2,8].

In planted forest ecosystems, changes in stand age significantly influence the distribution patterns and potential drivers of carbon stocks by altering vegetation structure, soil physicochemical properties, and environmental factors [9,10,11]. As stand age increases, the synergistic effects of processes such as tree-layer biomass accumulation, understory vegetation succession, litter input, and soil carbon pool stabilization give rise to complex carbon dynamics [12,13]. *Quercus acutissima* is a native hardwood species widely distributed across China’s subtropical to temperate regions. Characterized by rapid growth, strong adaptability, and tolerance to drought and poor soils, it is a dominant species for ecological restoration and a core species in the development of planted forests [14,15]. Its extensive cultivation area, approximately 8 million hectares, and significant carbon sink potential, accounting for 15.3% of the national planted forest area, highlight its important role in addressing global climate change and achieving carbon neutrality goals [14,15]. However, current research on *Q. acutissima* plantations has largely focused on directional cultivation and afforestation techniques for stands of a single age class, as well as the distribution patterns of carbon stocks, the effects of thinning or management density on carbon density, and changes in carbon stocks in mixed pine-oak ecosystems [14,16]. The dynamics and potential drivers of carbon pool distribution across different developmental stages remain insufficiently understood, particularly regarding the allocation patterns among vegetation, litter, and soil pools in fire-affected areas.

Therefore, this study selected *Q. acutissima* plantations of different stand ages (4, 10, 25, 45, and 50 years) on Huangfu Mountain as the study subjects. We systematically analyzed patterns of carbon stock change across ecological layers, including the tree layer, understory vegetation, litter layer, and soil layer, as a function of stand age. By quantifying the relationships between carbon stock dynamics and biological and environmental factors, we aimed to identify the main factors associated with carbon stock allocation patterns. Specifically, our primary objective was to investigate carbon storage distribution patterns in *Q. acutissima* plantations at five stand ages on the Huangfu Mountain fire-scarred site and to identify the main factors influencing carbon storage in these ecosystems. Therefore, this study formulates the following scientific hypotheses: (1) As forest age increases, carbon stocks in the tree canopy and soil layers increase significantly; (2) As forest age increases, the proportion of carbon stored in the soil layer decreases, but it remains the primary carbon pool; (3) Forest age regulates the carbon sequestration capacity of the *Q. acutissima* plantation ecosystem by influencing stand characteristics and soil factors. The findings of this study can provide a scientific basis for ecological restoration and for managing forest carbon sinks on fire-affected sites.

## 2. Materials and Methods

### 2.1. Study Area

The study area is located in the Huangfu Mountain State Forest Farm in eastern Anhui Province, China (117°58′–18°03′ E, 32°17′–32°25′ N), within the North Subtropical Humid Monsoon Climate Zone. Annual average precipitation exceeds 1000 mm, and the annual average temperature is approximately 14.7 °C. Rainfall is concentrated in the summer, particularly during the plum rain season, while winter precipitation is relatively low. Humidity is high, with an annual average humidity of around 70%. Due to the influence of the monsoon, the forest farm is primarily affected by northwesterly winds in winter and southeasterly winds in summer. The region has experienced large-scale, severe fires in the past. Since the 1970s, oak afforestation has been gradually implemented, with seedlings planted in the spring. Following afforestation, appropriate tending measures, such as weeding and fertilization, are carried out. Pruning is performed during the winter dormancy period (4–5 years), after which the area is managed under closed-forest conditions [14,16]. The study area has undergone significant ecological restoration following historical large-scale fires, primarily through the artificial establishment of *Q. acutissima* stands on fire-affected sites. As detailed in our previous work [14,16], the sampled stands represent a chronosequence of plantations established in 1973, 1978, 1998, 2013, and 2019. In this study, “stand age” refers to the plantation age, calculated as the time elapsed since the planting of *Q. acutissima* seedlings. These stands share comparable geological backgrounds and land-use histories. Uniform silvicultural management was applied across all sites, consisting of initial tending (e.g., fertilization and weeding) for the first 3–5 years post-planting, followed by natural growth without further intervention.

### 2.2. Plot Design

To represent different stages of forest development, five *Q. acutissima* plantations with essentially identical site conditions were selected, ranging in age from 4 to 50 years and covering the juvenile, middle-aged, near-mature, and mature growth stages of *Q. acutissima*. All selected plots were pure stands with canopy closure ≥ 0.75 and a dominant species basal area proportion ≥ 70%. Three 20 m × 20 m plots were established within each age-class plot, for a total of 15 plots. The distance between plots of different ages was at least 1 km to avoid spatial autocorrelation, and all plots were located at least 20 m from the forest edge to minimize edge effects [14,16].

### 2.3. Sample Collection

Ten trees were randomly selected from each plot, and samples of leaves, branches, stems, and roots were collected and thoroughly mixed [14]. For the shrub layer vegetation, three 5 m × 5 m subplots were randomly distributed within each plot of each forest age class, and the above- and below-ground biomass of shrubs and herbs within these subplots was harvested. A complete root harvesting method was employed for herbaceous plants. Soil was carefully excavated around the base of each target plant to collect all intact fine and coarse roots within the sampling range. The retrieved root systems were meticulously sorted to remove residual soil, necrotic roots, and other debris, thereby ensuring the integrity and purity of the samples. Subsequently, three 1 m × 1 m subplots were established within each plot across each stand to collect and weigh all litter within them. All collected plant materials, including tree trunks, branches, leaves, roots, and whole herbaceous plants, were transported to the laboratory. Previous studies on *Q. acutissima* plantations and pine-oak mixed forests have demonstrated that soil organic carbon (SOC) is predominantly concentrated in the topsoil and generally exhibits a decreasing trend with increasing soil depth [12,13]. Furthermore, topsoil properties are highly responsive to stand development and have been widely utilized to characterize soil variations across *Q. acutissima* plantations of different ages [14,15]. Therefore, the 0–30 cm soil layer was selected in this study to represent the carbon dynamics of the surface soil. Soil samples were collected, pooled, and homogenized from three randomly distributed points within each plot across each stand. Next, the humus layer was first removed from each point, and then the soil was dug to a depth of 30 cm with a hoe, dividing the soil profile into three layers (0–10 cm, 10–20 cm, 20–30 cm). Three ring-cutter samples (4 cm in diameter, 5 cm in height; Beijing Zhongke Luda Test Instrument Co., Ltd., Beijing, China) were collected from each soil layer. The composite samples were bagged, labeled, and sent to the laboratory for analysis.

### 2.4. Vegetation Survey Tree Biomass Estimation

In August 2024, tree height and diameter at breast height (DBH, cm) were measured for the tree layer vegetation within the plots. To assess the understory, three 5 × 5 m quadrats and ten 1 m × 1 m quadrats were systematically established in each plot to investigate the shrub and herb layers, respectively. For these layers, species identification and coverage were documented [17]. Plant diversity indices were calculated following the methodology proposed by Ma et al. and Fang et al. [14,18]. The tree biomass was estimated using allometric equations [19], and the specific formulas are as follows:

(1) $$W_{L}=0.0028{\left(D^{2}H\right)}^{0.8444}$$

(2) $$W_{Br}=0.0004{\left(D^{2}H\right)}^{1.3585}$$

(3) $$W_{S}=0.0235{\left(D^{2}H\right)}^{0.9792}$$

(4) $$W_{R}=0.0125{\left(D^{2}H\right)}^{0.9308}$$

where *W_L_*, $W_{Br}$, $W_{S}$, $W_{R}$ represents the leaf, branch, stem, and root biomass of each tree (kg), *D* denotes the DBH and *H* indicates the height of the trees (m).

(5) $$W_{leaf}=\sum_{k=1}^{n}W_{L,k}$$

(6) $$W_{branch}=\sum_{k=1}^{n}W_{Br,k}$$

(7) $$W_{stem}=\sum_{k=1}^{n}W_{S,k}$$

(8) $$W_{root}=\sum_{k=1}^{n}W_{R,k}$$

where *W_leaf_*, $W_{branch}$, $W_{stem}$, $W_{root}$ the leaf, branch, stem, and root biomass of the *k*-th tree (kg), and *n* is the total number of trees in the 400 m^2^ plot.

To standardize the biomass to a hectare scale (t ha^−1^), the total plot biomass (*W_leaf_*, $W_{branch}$, $W_{stem}$, $W_{root}$ was multiplied by the ratio of the standard area (10,000 m^2^) to the plot area (400 m^2^), and then converted from kilograms to tons:

(9) $$B_{leaf}=\frac{W_{leaf}\times 10000}{400\times 1000}=W_{leaf}\times 0.025$$

(10) $$B_{branch}=\frac{W_{branch}\times 10000}{400\times 1000}=W_{branch}\times 0.025$$

(11) $$B_{stem}=\frac{W_{stem}\times 10000}{400\times 1000}=W_{stem}\times 0.025$$

(12) $$B_{root}=\frac{W_{root}\times 10000}{400\times 1000}=W_{root}\times 0.025$$

where $B_{leaf},\;B_{branch},B_{stem},B_{root}$ is the *leaf*, branch, stem, root biomass (t ha^−1^).

### 2.5. Measurement of Substrate Diversity, Light Availability, and Heterogeneity

Within each 20 × 20 m plot, the areal coverage of exposed bedrock, litter, bare soil, uplifted root-associated soil, and coarse woody debris was estimated to calculate the substrate diversity index (SubD), which serves as an indicator of soil substrate heterogeneity. Furthermore, light availability and its spatial heterogeneity were assessed using a spherical densiometer (Model A, Forestry Suppliers, Inc., Jackson, MS, USA) at a height of 1.3 m above the ground in each herbaceous quadrat. Canopy openness (i.e., the percentage of sky not obscured by foliage) was measured in four cardinal directions (north, south, east, and west). The mean value of these measurements (LM) was used to represent light availability, while the standard deviation (LSD) was calculated to quantify light heterogeneity [17].

### 2.6. Sampling Analysis

After removing surface contaminants, the plant samples, including tree trunks, branches, leaves, roots and whole herbaceous plants, were cut into small pieces and oven-dried at 65 °C until a constant weight was achieved. The dried samples were then ground and passed through a 100-mesh sieve. Organic carbon (OC) content in each component was determined using the potassium dichromate external heating method. Finally, the carbon stock for each plant component was calculated by multiplying its dry biomass by the corresponding OC content. Soil bulk density was also determined using the oven-drying method, with soil samples dried to constant weight at 105 °C. Soil moisture content was measured by weighing the soil before and after drying, and calculated as the ratio of the dried soil weight to the original volume of the sampled soil. In the laboratory, samples of aboveground and belowground components, understory vegetation, leachates, and soil layers were dried, ground, and sieved, then bottled for chemical analysis. Organic carbon content was analyzed using the dichromate oxidation method and ferrous ammonium sulfate titration [20].

### 2.7. Carbon Sequestration Calculations

Upon determining the biomass and carbon content of the tree layer, understory vegetation (comprising shrubs and herbs), and litter, the carbon stocks were calculated using the following equations:

(1) Carbon stock of the tree layer:

(13) $$C_{leaf}=B_{leaf}\times S_{leaf}\times 10^{-3}$$

(14) $$C_{branch}=B_{branch}\times S_{branch}\times 10^{-3}$$

(15) $$C_{stem}=B_{stem}\times S_{stem}\times 10^{-3}$$

(16) $$C_{root}=B_{root}\times S_{root}\times 10^{-3}$$

(17) $$C_{tree}=C_{leaf}+C_{branch}+{C_{stem}+C}_{root}$$

where *C_tree_*, $C_{leaf}$, $C_{branch}$, $C_{stem}$, $C_{root}$ represents the carbon stock of the tree layer, *leaf*, branch, stem, root (t ha^−1^), $B_{leaf}$, $B_{branch}$, $B_{stem}$*,* $B_{root}$ denotes the *leaf*, branch, stem, root biomass (t ha^−1^), and $S_{leaf}$*_,_*$S_{branch}$, $S_{stem}$, $S_{root}$ indicates the organic carbon content of the *leaf*, branch, stem, root (g kg^−1^).

(2) Carbon stock of the understory vegetation (shrub and herb):

(18) $$C_{under}=B_{under}\times S_{under}\times 10^{-3}$$

where *C_under_* is the carbon stock of the understory vegetation (t ha^−1^), *B_under_* represents the understory biomass (t ha^−1^), and *S_under_* denotes the organic carbon content of the understory vegetation (g kg^−1^).

(3) Carbon stock of the litter layer:

(19) $$C_{litter}=B_{litter}\times S_{litter}\times 10^{-3}$$

where *C_litter_* stands for the carbon stock of the litter layer (t ha^−1^), *B_litter_* is the litter biomass (t ha^−1^), and *S_litter_* indicates the organic carbon content of the litter (g kg^−1^).

(4) Carbon stock of the soil layer:

(20) $$C_{soil}=0.1Hi\times {BD}_{soil}\times S_{soil}$$

where *C_soil_* represents the soil carbon stock (t ha^−1^), *Hi* is the thickness of soil layer *i* (cm), *BD_soil_* denotes the soil bulk density (g cm^−3^), and *S_soil_* is the soil organic carbon content (g kg^−1^).

(5) Ecosystem carbon stock:

(21) $$C_{Ecosystem}=C_{tree}+C_{under}+C_{litter}+C_{soil}$$

where *C_Ecosystem_*, *C_tree_*, *C_under_*, *C_litter_*, and *C_soil_* represent the carbon stocks of the ecosystem, tree layer, understory vegetation, litter layer, and soil layer, respectively (t ha^−1^) [21].

### 2.8. Statistical Analysis

One-way analysis of variance (ANOVA) was used to analyze carbon stocks across components of *Q. acutissima* plantation ecosystems at different ages, with post hoc tests conducted using the Least Significant Difference (LSD) method. Bartlett’s test and Shapiro’s test were used to verify the assumptions of homoscedasticity and normality. Correlation analysis and Mantel tests were used to examine relationships between carbon stocks across ecosystem components and environmental variables, including plant diversity, soil properties, and bacterial/fungal diversity. Random forests were employed to identify key factors influencing ecosystem carbon stocks, and a prior model was established. Subsequently, the “lavaan” package was used to construct and analyze structural equation models (SEM). Given the limited number of study plots in this research, random forest and structural equation models were primarily used to explore potential associations between different stand structures, soil properties, and environmental factors and carbon stocks, rather than to validate strict causal mechanisms. All analyses were conducted using R software (V4.2.3, R Foundation for Statistical Computing, Vienna, Austria) [14,22].

To verify the reliability and regional applicability of the study’s results, a comparative analysis of the literature was conducted to integrate this study’s data with published data on ecosystem carbon stocks from *Q. acutissima* and related broadleaf tree species, performing trend fitting and cross-comparisons. Through a literature search covering the period from 2015 to 2026, using the keywords “planted forests, ecosystem carbon stocks, and age-class sequences” (in the China National Knowledge Infrastructure [CNKI] and Web of Science databases), more than 100 relevant articles were identified. These were then screened based on the principles of consistency in study areas, stand types, measurement methods, and indicator systems. Through a literature search and subsequent screening, we included data from studies on *Q. acutissima*, *Castanopsis hystrix*, *Castanea mollissima* Blume (chestnut), and other similar species. Using forest age as the core variable, we extracted ecosystem carbon stock indicators to compare trends and validate patterns, thereby assessing the general applicability of our findings. All statistical analyses and graphical representations were performed in R software (V4.2.3, R Foundation for Statistical Computing, Vienna, Austria).

## 3. Results

### 3.1. Carbon Stocks in Various Components of Q. acutissima

**Figure 1:**
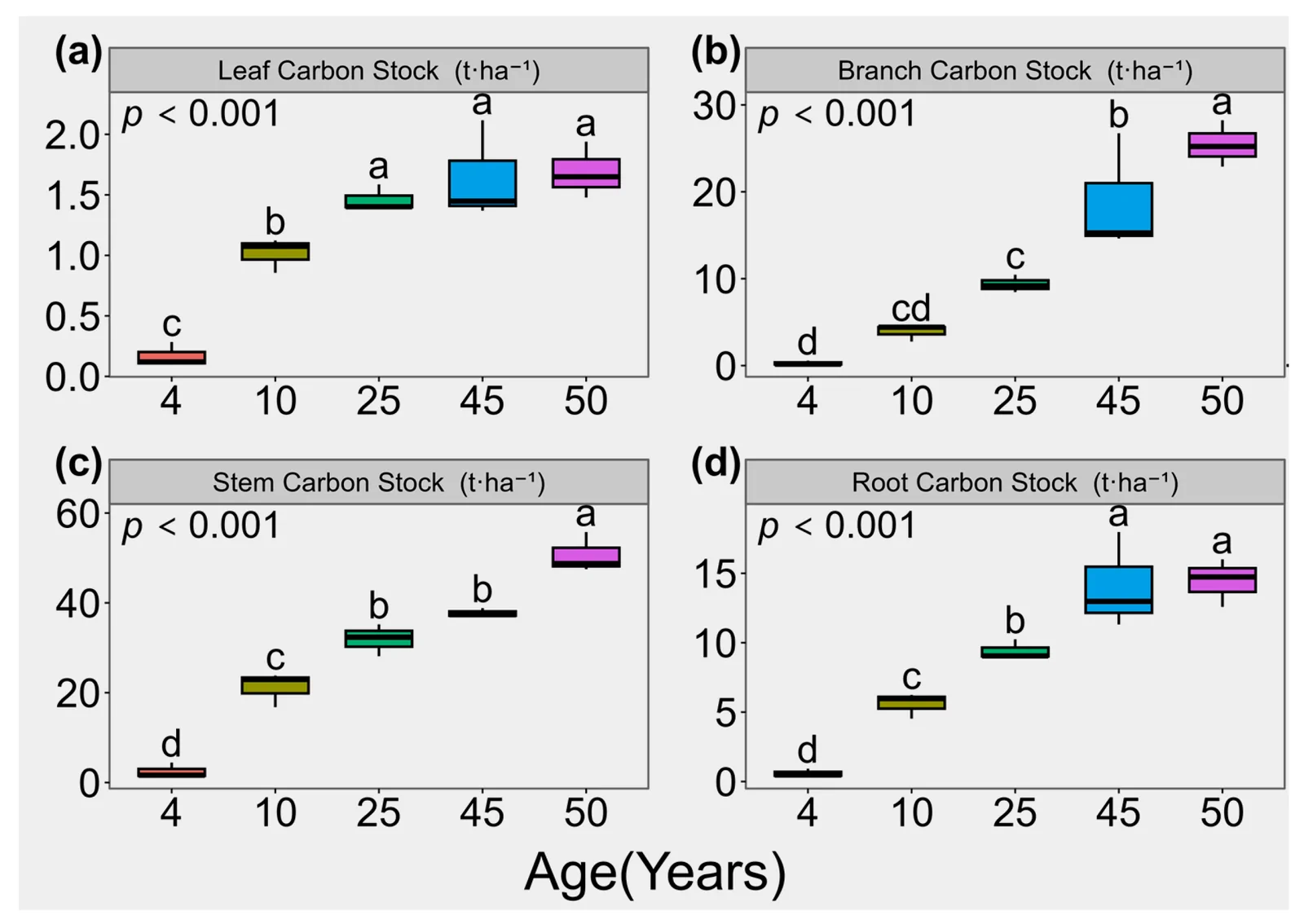
Dynamic changes of carbon stocks along the planting age gradient in different tree components of *Quercus acutissima* plantations. Panels show carbon stocks in (**a**) leaves, (**b**) branches, (**c**) stems, and (**d**) roots, expressed as t ha^−1^. Different lowercase letters indicate significant differences among stand ages at *p* < 0.05.

The study found a positive correlation between carbon stocks in various tree organs and stand age (Figure 1). Among all tree components, carbon stocks in leaves (*p* < 0.001), branches (*p* < 0.001), stems (*p* < 0.001), and roots (*p* < 0.001) increased significantly with increasing stand age (Figure 1). Among the components of *Q. acutissima* stands, stem carbon stocks were the highest (*p* < 0.001), amounting to 2.48 t ha^−1^, 21.17 t ha^−1^, 31.90 t ha^−1^, 37.70 t ha^−1^, and 50.67 t ha^−1^.

### 3.2. Dynamic Changes of Carbon Stocks Along the Planting Age Gradient in Different Soil Layers

**Figure 2:**
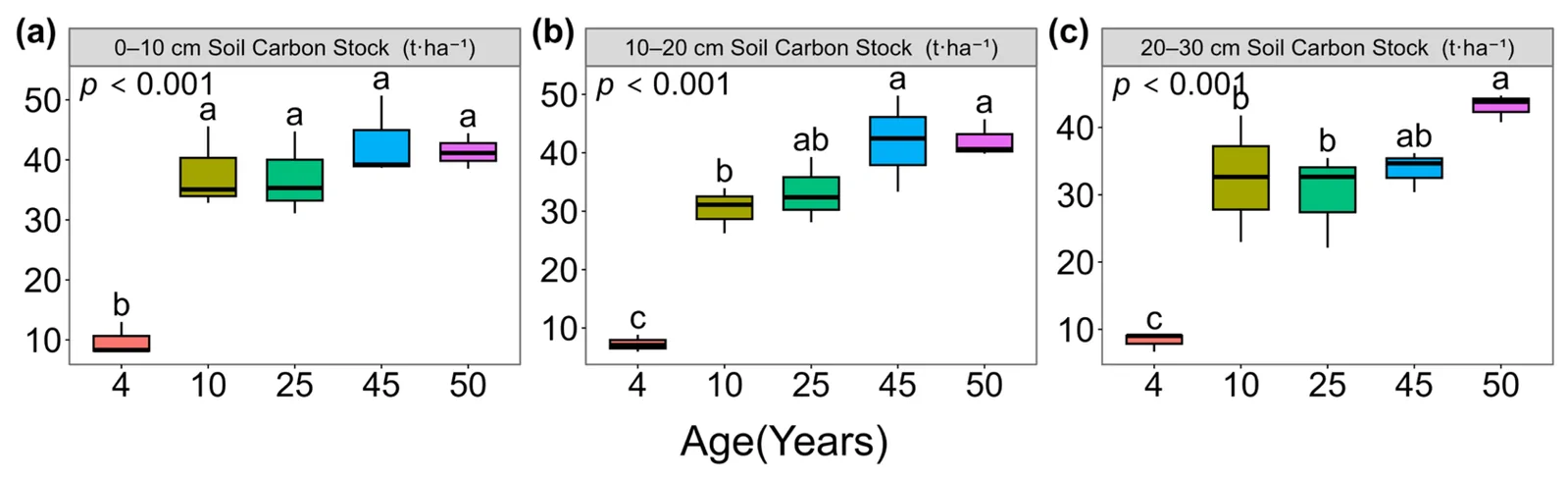
Dynamic changes of carbon stocks along the planting age gradient in *Quercus acutissima* plantations. Panels show soil carbon stocks at different soil depths: (**a**) 0–10 cm, (**b**) 10–20 cm, and (**c**) 20–30 cm, expressed as t ha^−1^. Different lowercase letters indicate significant differences among stand ages at *p* < 0.05.

As shown in Figure 2, soil carbon stocks generally decreased with increasing soil depth, indicating that carbon accumulation was concentrated mainly in the surface soil (0–10 cm). Total soil carbon stocks in the 0–30 cm soil layer showed a significant increasing trend among stands aged 4–50 years, ranging from 25.32 t ha^−1^ to 126.56 t ha^−1^.

### 3.3. Carbon Storage in the Quercus acutissima Plantation Ecosystem and Its Ecological Components

**Figure 3:**
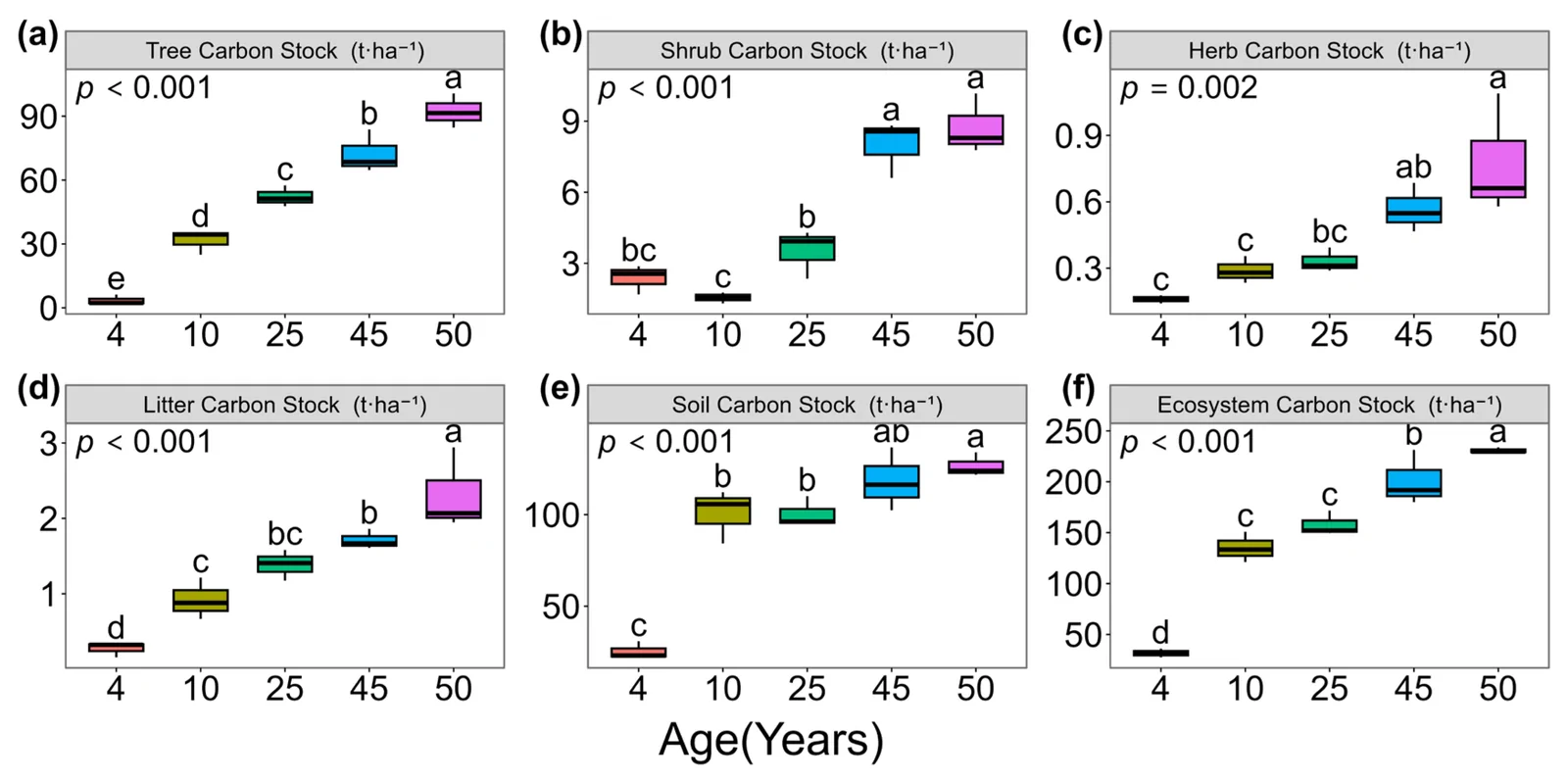
Effects of stand age on carbon stocks across ecosystem components in *Quercus acutissima* plantations. Panels show carbon stocks in (**a**) tree layer, (**b**) shrub layer, (**c**) herb layer, (**d**) litter layer, (**e**) soil layer, and (**f**) total ecosystem, expressed as t ha^−1^. Different lowercase letters indicate significant differences among stand ages at *p* < 0.05.

With increasing stand age, significant increases in carbon storage were observed in the herb layer (*p* = 0.002) and the litter layer (*p* < 0.001) (Figure 3). However, the carbon storage of the shrub layer showed an initial decline followed by an increase with increasing stand age, reaching a maximum of 8.75 t ha^−1^ in 50-year-old stands. In 4-year-old and 10-year-old stands, the proportion of carbon storage in the herb layer was relatively small, accounting for only 0.14% and 0.28%, respectively. The carbon storage in the arbor layer increased significantly with stand age, reaching 3.52 t ha^−1^ in 4-year-old stands and 92.25 t ha^−1^ in 50-year-old stands (Figure 3).

The results show that the soil layer is the largest carbon pool in this ecosystem, with carbon storage higher than that of all components of *Q. acutissima* trees, litter, and understory plants combined. The soil layer is the dominant carbon pool in the total ecosystem of each stand, with carbon storage of 25.32 t ha^−1^, 100.72 t ha^−1^, 100.41 t ha^−1^, 118.47 t ha^−1^, and 126.56 t ha^−1^ in 4-year-old, 10-year-old, 25-year-old, 45-year-old, and 50-year-old stands, respectively. The total carbon storage of the *Q. acutissima* plantation ecosystem increased significantly with increasing stand age (*p* < 0.001), with values of 31.64 t ha^−1^, 135.12 t ha^−1^, 157.81 t ha^−1^, 201.06 t ha^−1^, and 230.66 t ha^−1^ in 4-year-old, 10-year-old, 25-year-old, 45-year-old, and 50-year-old stands, respectively (Figure 3).

### 3.4. Key Factors Affecting Ecosystem Carbon Stocks in Q. acutissima Plantations

**Figure 4:**
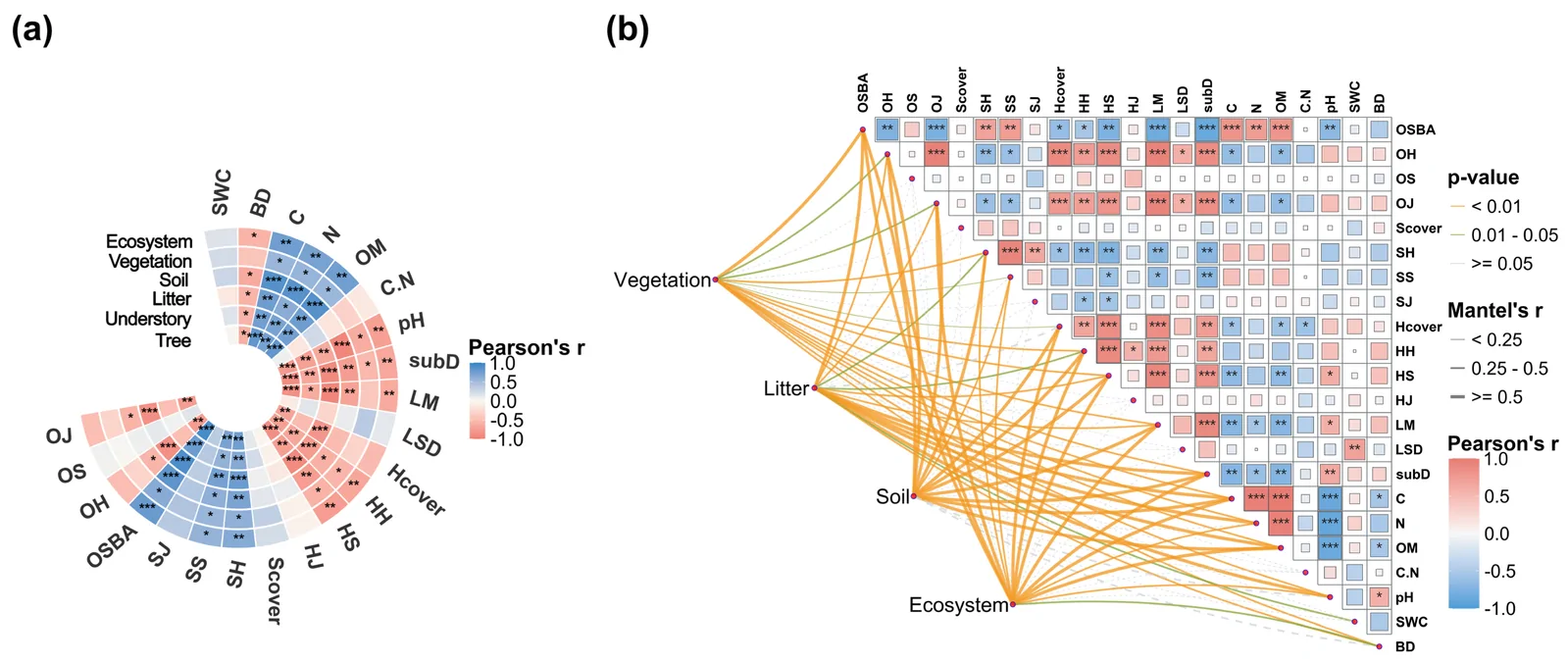
Relationships between ecosystem carbon stocks and biotic and abiotic factors in *Quercus acutissima* plantations. (**a**) Correlation heatmap shows associations among carbon stocks, stand structure, vegetation diversity, light conditions and soil properties, indicators; (**b**) Mantel test analysis showing the correlations between environmental factors and community structure. Variable abbreviations are as follows: tree stem basal area (OSBA), tree Shannon index (OH), tree species richness (OS), tree evenness (OJ), shrub cover (Scover), shrub Shannon index (SH), shrub species richness (SS), shrub evenness (SJ), herb cover (Hcover), herb Shannon index (HH), herb species richness (HS), herb evenness (HJ), light availability (LM), light heterogeneity (LSD), substrate diversity (subD), soil carbon (C), soil nitrogen (N), soil organic matter content (OM), soil carbon-to-nitrogen ratio (C/N), soil pH (pH), soil water content (SWC), soil bulk density (BD), vegetation carbon stock (Vegetation), litter carbon stock (Litter), soil carbon stock (Soil), and ecosystem carbon stock (Ecosystem), * *p* < 0.05, ** *p* < 0.01, *** *p* < 0.001. Orange and green lines denote significant and non-significant correlations, respectively.

As shown in Figure 4, ecosystem carbon stock was significantly positively correlated with shrub richness, the shrub Shannon index, tree basal area, soil organic matter content, soil nitrogen, and soil carbon; and was significantly negatively correlated with substrate diversity, soil pH, tree evenness, the tree Shannon index, light availability, herb richness, the herb Shannon index, herb cover, and soil bulk density. Vegetation carbon stock was significantly positively correlated with shrub richness, shrub Shannon index, tree breast-height cross-sectional area, soil organic matter content, soil nitrogen, and soil carbon; it was significantly negatively correlated with substrate diversity, soil pH, light availability, tree Shannon index, tree evenness, herbaceous cover, herbaceous richness, herbaceous Shannon index, and soil bulk density. Soil carbon stock was significantly positively correlated with shrub richness, shrub Shannon index, tree breast-height cross-sectional area, soil organic matter content, soil nitrogen, and soil carbon; it was significantly negatively correlated with substrate diversity, soil pH, tree evenness, tree Shannon index, light availability, herbaceous richness, herbaceous Shannon index, herbaceous cover, and soil bulk density. Litter carbon stock was significantly positively correlated with shrub richness, shrub Shannon index, tree basal area, soil organic matter content, soil nitrogen, and soil carbon; it was significantly negatively correlated with substrate diversity, soil pH, tree evenness, tree Shannon index, light availability, herbaceous richness, herbaceous Shannon index, and soil bulk density. Understory vegetation was significantly positively correlated with shrub richness, the Shannon index of shrubs, tree basal area, soil organic matter content, soil nitrogen, and soil carbon, and was significantly negatively correlated with substrate diversity, soil pH, herbaceous richness, and the Shannon index of herbs. Carbon stock in the tree layer was significantly positively correlated with shrub richness, the Shannon index of shrubs, tree basal area, soil organic matter content, soil nitrogen, and soil carbon, and was significantly negatively correlated with substrate diversity, soil pH, light availability, herbaceous richness, the Shannon index of herbs, and soil bulk density.

### 3.5. Regulatory Influencing Factors of Carbon Stock in Q. acutissima Plantations

**Figure 5:**
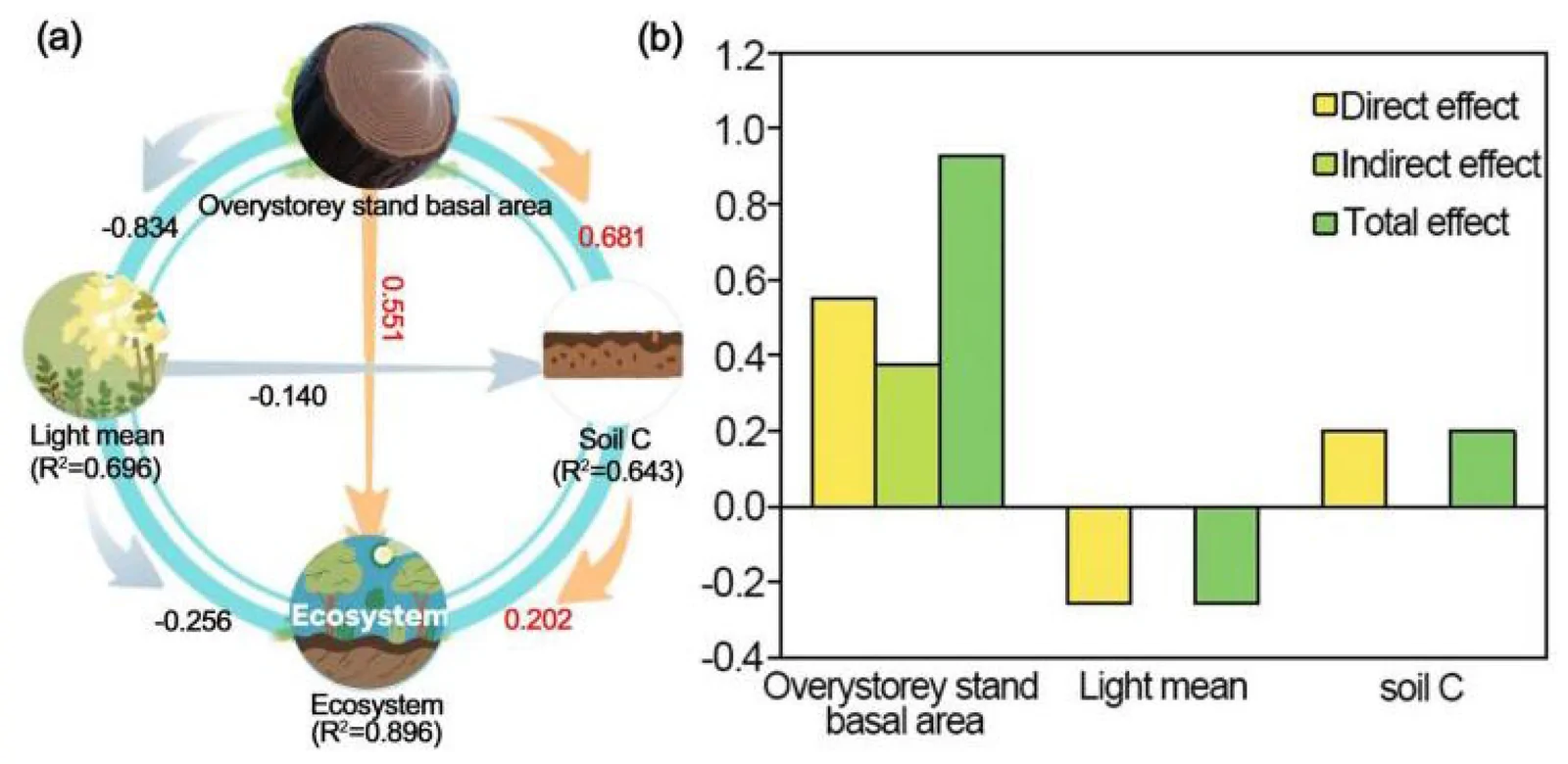
Regulatory influencing factors of carbon stock in *Quercus acutissima* plantations. (**a**) Structural equation model (SEM) showing the direct and indirect pathways affecting ecosystem carbon stock. Solid orange and gray arrows indicate significant positive and negative effects, respectively (*p* < 0.05). The numbers next to the variables represent the proportion of variance explained by the model (R^2^), and the numbers on the arrows represent the standardized path coefficients. (**b**) Decomposition of direct, indirect, and total effects of overstorey stand basal area, light mean, and soil C on ecosystem carbon stock derived from the SEM.

Structural equation modeling results indicate that the breast-height cross-sectional area of trees has a direct effect on the ecosystem carbon stock index (0.551) and an indirect effect via light availability and soil carbon (0.375). Light availability has a direct negative effect on the ecosystem carbon stock index (−0.256). The study found that 89.6% of the variation in the ecosystem carbon stock index is explained by breast-height tree cross-sectional area, light availability, and soil carbon (Figure 5).

### 3.6. A Comparative Analysis of Ecosystem Carbon Stocks in Q. acutissima and Other Oak Species and Similar Tree Species

**Figure 6:**
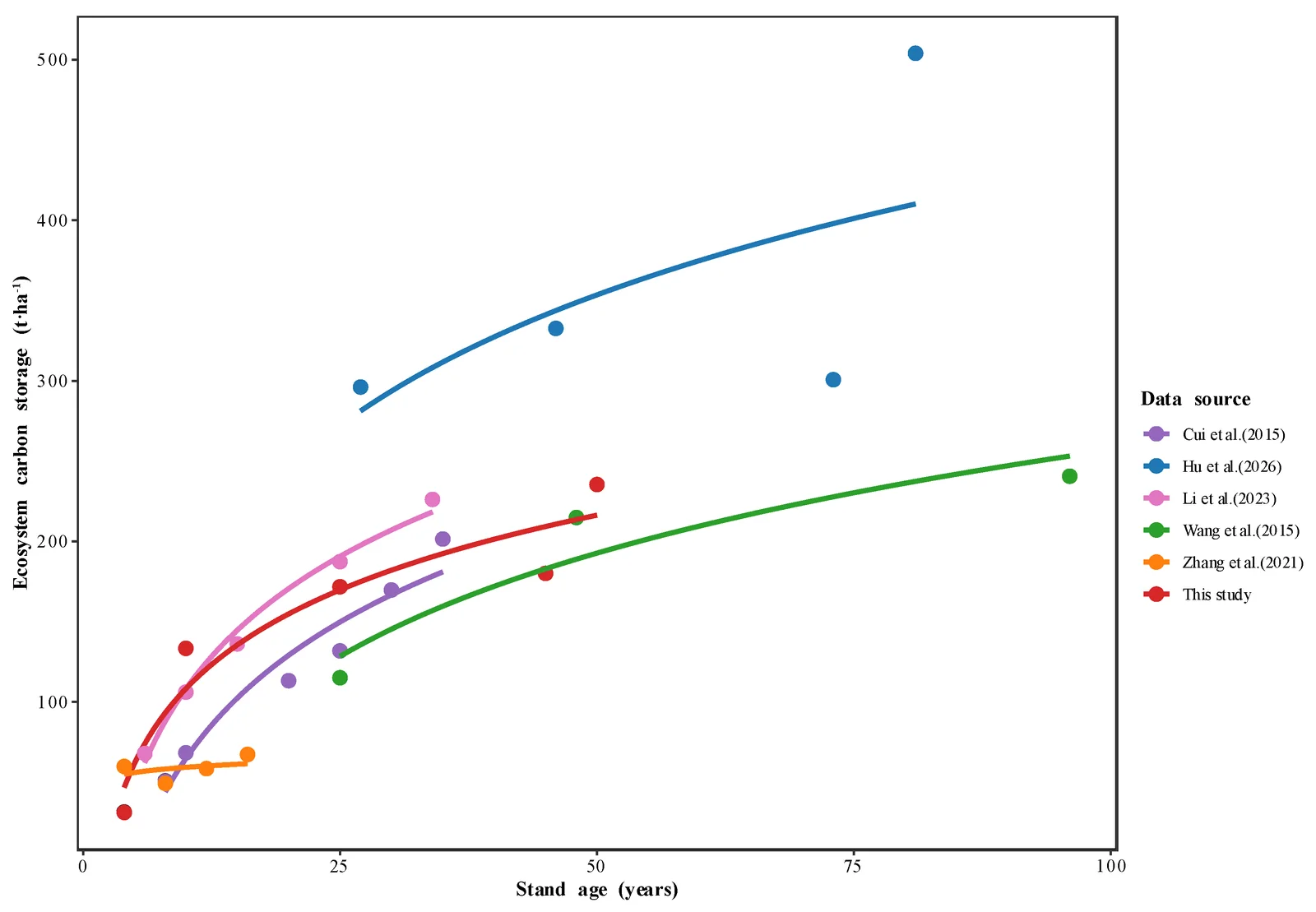
Comparative analysis of ecosystem carbon stocks in *Quercus acutissima* and other oak or similar broadleaf tree species. The figure compares ecosystem carbon stocks across stand ages using data from the present study and previously published studies [13,21,23,24,25].

To verify the reliability of the patterns of carbon stock changes in the *Q. acutissima* plantation ecosystem on fire-affected sites identified in this study, and given the current limited body of research on *Q. acutissima*, we conducted a cross-study comparative analysis of the results from this study with published data on carbon stocks of the genus *Quercus* [13,23,24] and subtropical closely related broadleaf tree species [21,25] (Figure 6). The included studies covered research on the genera *Quercus* and *Castanopsis*, including *Castanopsis hystrix*, and chestnut trees.

As shown in Figure 6, ecosystem carbon stocks for both the genus *Quercus* and similar tree species exhibit an upward trend with increasing stand age, and the patterns of change are generally consistent across different studies. The carbon stocks of Chinese oak at different stand ages in this study fall within the ranges reported in similar studies; carbon stocks gradually increase with the prolongation of the recovery period, showing a trend similar to that of species such as *Quercus*, *Castanopsis hystrix*, and chestnut. Carbon stocks in *Q. acutissima* stands on fire-scarred sites are low during the early stages of recovery but accumulate rapidly as the stand develops, approaching the carbon stock levels of mature stands in similar regions by the 50th year.

The results indicate that stand age is a key driver of ecosystem carbon accumulation, while site conditions, fire disturbance, and stand structure account for variation in values across studies. The findings of this study are consistent with the patterns of carbon sink dynamics in regional broadleaf tree species, demonstrating good representativeness and reliability.

## 4. Discussion

This study examined the distribution patterns of ecosystem carbon stocks and the potential factors associated with different stages of artificial restoration following severe forest fires. The results indicate that stand age is a key factor associated with carbon storage dynamics in *Q. acutissima* plantations, with significant differences in carbon stock distribution among trees, understory vegetation, litter, and soil at different stand ages [21,26]. As stand age increased, the total carbon stock of the *Q. acutissima* plantation ecosystem increased significantly, from 31.64 t ha^−1^ in 4-year-old stands to 230.66 t ha^−1^ in 50-year-old stands. This trend was closely linked to the accumulation of tree biomass, increased litter input, and the gradual development and stabilization of soil carbon pools [27,28]. These results suggest that carbon recovery in fire-affected *Q. acutissima* plantations is not controlled by a single component, but rather involves the coordinated development of aboveground vegetation, litter return, and belowground carbon accumulation. Therefore, stand age should be considered an important factor when evaluating carbon sink recovery and forest restoration effectiveness in post-fire plantations.

Among the different tree organs, stem carbon accounted for the largest proportion of tree-layer carbon storage and increased significantly with stand age. This pattern is closely related to the biological characteristics of *Q. acutissima*. As a hardwood species, *Q. acutissima* gradually accumulates woody biomass during stand development, and stems serve as the main long-term carbon storage organ [29]. The significant increases in leaf, branch, stem, and root carbon stocks shown in Figure 1 further indicate that tree-layer carbon accumulation occurs through the coordinated growth of multiple organs, with stem biomass contributing most strongly to long-term carbon storage. Furthermore, tree basal area, as an important stand structural variable, was closely associated with ecosystem carbon storage. Structural equation modeling showed that tree basal area had a direct association with ecosystem carbon stock (standardized path coefficient = 0.551) and an indirect link through light availability and soil carbon (path coefficient = 0.375). This suggests that tree basal area may reflect ecosystem carbon storage both by representing woody biomass accumulation and by being linked to canopy structure, understory light conditions, litter input, and soil carbon accumulation. This finding is consistent with previous studies showing that tree-layer structural characteristics play an important role in forest carbon sink function [30,31,32].

The soil layer was the largest carbon pool in the *Q. acutissima* plantation ecosystem, and soil carbon stocks increased with stand age, especially in the 0–10 cm topsoil layer [33,34]. Soil carbon stocks increased rapidly between 4 and 10 years but remained virtually unchanged between 10 and 25 years. This change may indicate that surface soil carbon accumulation has entered a short-term stabilization phase during stand development, which may be related to litter input, root turnover, decomposition processes, and differences in soil properties [5,13,15]. As shown in Figure 2, soil carbon stocks generally decreased with increasing soil depth, indicating that carbon accumulation was concentrated mainly in the surface soil. This pattern may be explained by the continuous input of litter and fine roots during stand development. As plantations mature, increased canopy biomass produces more litter, while root turnover provides additional organic matter belowground. These organic inputs are first incorporated into the upper soil layer, leading to stronger carbon accumulation in the 0–10 cm soil depth than in deeper layers. In addition, soil organic matter, soil carbon, and soil nitrogen were positively correlated with ecosystem carbon stocks, whereas soil bulk density and pH were negatively correlated. These relationships suggest that soil fertility and soil physical conditions are important for maintaining carbon accumulation in *Q. acutissima* plantations. Therefore, improving soil organic matter accumulation and maintaining favorable soil structure may be important strategies for enhancing carbon sequestration capacity in these fire-affected plantations.

Although carbon stocks in the understory vegetation and litter layers accounted for a relatively small proportion of total ecosystem carbon, their dynamic changes reflected the process of stand succession. As shown in Figure 3, litter carbon stocks increased significantly with stand age, indicating that litter input increased as the tree layer developed [10,11]. The litter layer serves as an important transitional carbon pool between vegetation and soil because litter decomposition transfers part of plant-derived carbon into soil organic matter. Shrub carbon stocks reached their highest level in 50-year-old stands, suggesting that shrub-layer development became more pronounced in older plantations. Herbaceous-layer carbon stocks were more strongly affected by light availability and substrate conditions [14,17]. The structural equation model showed that light availability had a direct negative association with ecosystem carbon stock, with a standardized path coefficient of −0.256. This negative relationship likely reflects the fact that younger or more open stands have higher light availability but lower tree biomass, litter input, and soil carbon accumulation. In contrast, older stands generally have greater canopy closure, lower understory light availability, and higher ecosystem carbon stocks [14,17]. Thus, this negative path reflects the covariation between canopy density and carbon stocks rather than a direct negative effect of sunlight itself on carbon accumulation. These variables are closely related to changes in ecosystem carbon stocks, thereby reflecting the combined effects of stand structure, soil conditions, and canopy development on carbon pool distribution.

Through comparative analysis, this study examined the dynamics of carbon stocks in fire-affected *Q. acutissima* plantations and in other *Quercus* species, *Castanopsis hystrix*, and *Castanea* species. The results showed that ecosystem carbon stocks in these studies generally increased with stand age. The carbon stock values observed in the present study fell within the range reported for similar studies, and the overall pattern of change was broadly consistent [21,27]. Although differences among studies may be due to variation in site conditions, fire disturbance intensity, stand structure, management history, and soil properties, the overall trend of increasing carbon accumulation with stand age remained clear. As shown in Figure 6, carbon stocks in *Q. acutissima* plantations were relatively low during the early restoration stage but increased rapidly during stand development, approaching the carbon stock levels reported for mature broadleaf plantations in similar regions [21,27]. These results indicate that *Q. acutissima* plantations on fire-affected sites have considerable potential to recover carbon sinks.

In summary, this study systematically analyzed the distribution patterns and factors associated with carbon stocks in *Q. acutissima* plantations of different ages. The results are consistent with those of Song et al. [12] and Cui et al. [13], which showed that total carbon stocks in plantation ecosystems increase significantly with stand age and that the soil layer is a major contributor to ecosystem carbon storage. However, regarding the vertical distribution of soil carbon, this study found that carbon accumulation was most pronounced in the 0–10 cm topsoil layer. This result is similar to the conclusions of Tao et al. [33] for *Pinus massoniana* plantations but differs from those of Wei et al. [34] for other stand types. This difference may be related to variation in litter input, litter decomposition rate, root distribution, and soil microbial activity among different plantation types. Regarding the key factors influencing carbon stocks, this study suggests that tree basal area plays a central role in carbon sink function. This is consistent with the findings of Cheng et al. [29] on carbon density in oak plantations and those of Feng et al. [35] on carbon stocks in spruce plantations, both of which emphasized the importance of tree-layer structure in forest carbon storage. Moreover, this study further quantified the direct association of tree basal area with ecosystem carbon stock and its indirect link through light availability and soil carbon using structural equation modeling. This helps explain how stand development may be associated with ecosystem carbon accumulation through both biomass accumulation and soil carbon pathways. Furthermore, the negative correlation of light availability with carbon stocks differs from the findings of Liu et al. [27] in poplar plantations, which may reflect differences in canopy development, stand structure, and light-use responses among tree species. Regarding soil carbon regulation, this study supports the conclusion of [15] that soil carbon, nitrogen, and organic matter contents are positively correlated with ecosystem carbon stocks, but differ from the findings of [16], likely due to differences in litter input, soil properties, and microbial decomposition efficiency among different stand types [36,37,38]. Furthermore, the negative correlations of soil bulk density and pH with carbon stock are consistent with the study of soil properties in *Q. acutissima* plantations by Wei et al. [34], further indicating that soil physical and chemical conditions can influence carbon sequestration capacity [36,39,40,41].

Overall, the findings demonstrate that ecosystem carbon stocks in fire-affected *Q. acutissima* plantations increase substantially with stand age. The recovery process is mainly associated with tree biomass accumulation, litter input, and soil carbon development. Tree basal area, soil organic matter, soil nitrogen, and soil carbon were closely related to ecosystem carbon stocks, while light availability reflected changes in canopy closure during stand development [36,39,40,41]. These results provide useful evidence for understanding post-fire carbon recovery in *Q. acutissima* plantations and suggest that forest management should focus on promoting healthy stand growth dynamics and maintaining soil quality to enhance long-term carbon sink function [39,40,41,42,43].

This study included a total of 15 plots; the limited sample size may affect the stability and inferential power of the multivariate statistical analyses. Therefore, the results of the correlation analysis, random forest analysis, and exploratory structural equation modeling should be interpreted as potential associations rather than definitive causal relationships. Additionally, as soil carbon was measured only to 30 cm depth, the conclusions regarding soil carbon stocks represent surface soil dynamics rather than the full soil profile. Future research should combine long-term fixed-site monitoring, a larger plot network, sampling of deeper soil layers, and a more complete reconstruction of fire history to further validate the carbon recovery patterns observed in this study.

## 5. Conclusions

Carbon stocks in *Q. acutissima* plantations established on fire-affected sites increased significantly with stand age across different recovery stages, rising from 31.64 t ha^−1^ in 4-year-old stands to 230.66 t ha^−1^ in 50-year-old stands. The surface soil layer (0–30 cm) represented the largest carbon pool in these plantation ecosystems, with carbon stocks accumulating progressively with stand age, particularly in the 0–10 cm layer. Stand age emerged as an important factor associated with the distribution pattern of carbon stocks in *Q. acutissima* plantations. Tree basal area was strongly linked to ecosystem carbon sequestration through potential direct and indirect pathways involving light availability and soil carbon, while soil organic matter and nitrogen content showed significant positive correlations with carbon stocks. Different stand-age stages exhibited distinct carbon allocation characteristics: young stands were characterized by rapid tree-layer carbon accumulation, whereas mature stands were more strongly associated with soil carbon accumulation and stabilization. In future forest management, emphasis should be placed on optimizing stand density and maintaining appropriate light conditions through measures such as thinning, while simultaneously strengthening soil fertility management to enhance the carbon sink potential of *Q. acutissima* plantations. Furthermore, site selection for afforestation should prioritize loose, well-aerated sandy or sandy loam soils and avoid poorly aerated heavy clay soils to promote the healthy growth and carbon accumulation of *Q. acutissima*. This study provides evidence for improving the sustainable management and carbon sequestration capacity of *Q. acutissima* plantations on fire-affected sites, with implications for forest restoration and carbon-neutrality strategies.

## Data Availability

The raw data supporting the conclusions of this article will be made available by the authors on request.
